# Whole genome sequence analysis of two subspecies of *Companilactobacillus Futsaii* and experimental verification of drug resistance and effect on the exploratory behavior of mice based on unique gene

**DOI:** 10.1371/journal.pone.0274244

**Published:** 2022-09-09

**Authors:** Zhao Xin, Xing Wei, Qiuxia Jiao, Qiufeng Gou, Yumeng Zhang, Chaoming Peng, Qu Pan

**Affiliations:** 1 Department of Pathogenic Biology, Chengdu Medical College, Chengdu, China; 2 Department of Clinical Laboratory, Pidu District People’s Hospital, Chengdu, China; 3 First Affiliated Hospital, Chengdu Medical College, Chengdu, China; University of Nebraska-Lincoln, UNITED STATES

## Abstract

This study characterized the whole genome of *Companilactobacillus futsaii* subsp. *chongqingii* CQ16Z1 isolated from Chongqing of China, performed genome sequence analysis with *Companilactobacillus futsaii* subsp. *futsaii* YM0097 isolated from Taiwan of China, and experimentally verified drug resistance and effect on the exploratory behavior of male C57BL/6 mice and analysis of gut microbiota and metabolomic studies. The genome of CQ16Z1 is 2.6 Mb. Sequence analysis between genomes showed that the two strains are *Companilactobacillus futsaii*. The unique genes of CQ16Z1 and YM0097 are 217 and 267, which account for 9% and 11% of the whole genomes, respectively. According to unique gene annotation, the results showed that genes associated with carbohydrate metabolism, environmental information processing, metabolism of cofactors and vitamins, cell wall/membrane/envelope biogenesis, phage and drug resistance are significantly different. The results of the drug resistance experiment showed that YM0097 had different degrees of resistance to 13 antibiotics, while CQ16Z1 was sensitive to more than half of them. YM0097 contains 9 prophage regions and CQ16Z1 contains 3 prophage regions. The results of the open field test showed that the time (*P* = 0.005; *P* = 0.047) and distance (*P* < 0.010; *P* = 0.046) of the central area of Y97 group and CQ group are significantly different from the control group. The results of the elevated plus maze test showed that compared with the control group, Y97 group had significant differences in the number of entries to the open arms and the percentage of open arms entry times (*P* = 0.004; *P* = 0.025), while the difference between the CQ group and the control group was not significant. YM0097 has a more obvious effect on the exploratory behavior of mice. The effects of YM0097 and CQ16Z1 on the intestinal flora of mice are also different. YM0097 may be more beneficial to the intestinal flora of the host. And LC/MS also showed that the metabolic effects of the two strains on the host are different. Finally, we believe that YM0097 is more suitable for application research as a psychobiotics.

## Introduction

*Lactobacillus* is a facultative-anaerobic Gram-positive rod-shaped bacterium that fermentally utilize carbohydrates and take lactic acid as the final product [1]. It is a safe and harmless microorganism, which is widely distributed on the surface of plants, traditional fermented food, dairy products and human and animal intestines [2]. Due to its large number of members and great differences, *lactobacillus* has been divided into 25 genera recently [3]. *Companilactobacillus futsaii* is a new species isolated from Taiwan [4]. Other strain of *Companilactobacillus futsaii* identified in the fermented products of Thailand can be used as the production strain of γ-aminobutyric acid [5]. In 2019, we completed the whole genome sequencing of *Companilactobacillus futsaii* subsp. *futsaii* YM0097^T^ (= JCM17355^T^ = BCRC80278^T^) [6] (hereinafter referred to as Y97). CQ16Z1 was isolated by our team from jamiecosley, a traditional pickle, commonly homemade in Chongqing, and identified as a new subspecies of *Companilactobacillus futsaii*, named *Companilactobacillus futsaii* subsp. *chongqingii* subsp.nov, CQ16Z1^T^ (= CCTCCAB 2017187^T^ = KCTC 21089^T^) [7] (hereinafter referred to as CQ16Z1).

With the development of second and third generation sequencing technologies, sequence analysis between genomes has been widely used in the study of microbial genomes. High throughput sequencing analysis of samples based on the V3-V4 variable region of 16S rRNA gene is helpful to further study microbial populations. In addition, the use of metabolomics through the use of LC/MS can provide insights into disease progression through the detection of disease-associated metabolites. Examples of these metabolites include cortisol, 4-hydroxybenzoic acid, 5-hydroxyindole-3-acetic acid, kynurenic acid, 3-indoleacrylic acid and hippuric acid which are biomarkers of depression and/or anxiety [8–11].

Probiotics have been widely reported which provides health benefits to the host through interacting with intestinal cells or the intestinal microbiome [12]. Dinan and others believed that the regulation of the host’s gut microbiome may be an effective strategy for the treatment of mental illness [13]. *Lactobacillus* and *Bifidobacteria* can achieve a positive impact on the host’s mental health and brain function through the brain-gut axis, and are called psychobiotics [14]. At present, *Lactobacillus* and *Bifidobacterium* are the main psychobiotics which have shown beneficial effects in clinical treatment, such as improving patients’ anxiety and cognition [15,16]. In addition, in animal experiments, *Lactobacillus plantarum* also has obvious probiotic properties. Study showed that the cognition of male mice can be improved by *Lactobacillus plantarum 286* [17]. Although probiotics have many benefits, their safety has gradually attracted people’s attention. Study showed that some probiotics may have adverse effects on host health, such as immunosuppressive status and antibiotic resistance gene transfer [18]. The transfer of antimicrobial resistance (AMR) genes increases the risk of antibiotic resistance treatment in bacteria, and these issues require extensive attention.

This study reported the whole genome of CQ16Z1, and revealed the whole genome difference between it and Y97. In addition, strain Y97 displayed greater beneficial effects on mice behavior, intestinal flora and serum metabolites compared to CQ16Z1, suggesting its potential use as a probiotic that is beneficial for host health.

## Materials and methods

### Experimental strains and culture

CQ16Z1 was preserved by the Microbiology Laboratory of Chengdu Medical College. Y97 was obtained from the Bioresource Collection and Research Center (BCRC, Taiwan, China). CQ16Z1 was cultured in MRS liquid medium at 37°C for 18 hours, then the bacterial solution was sub packed into a test tube in a biosafety cabinet (Thermo Fisher Scientific, USA), centrifuged at 13000 g for 1 min. The bacteria were sent to Wuhan Frasergen Information Co., Ltd. for whole genome sequencing.

### CQ16Z1 sequencing, assembly and annotation

The whole genome of CQ16Z1 was sequenced, assembled and annotated in combination with second-generation Illumina sequencing and third-generation Nanopore sequencing technology. The experimental process was performed in accordance with the standard protocol provided by Oxford Nanopore Technologies (ONT), including sample quality detection, library construction, library quality detection and library sequencing. After sequencing, the original data of Nanopore sequencing and the raw data produced by Illumina second-generation sequencing were evaluated for quality. Unicycler [19] (version: 3.0) was used to assemble the filtered illumina data (Q30 > 85%) to obtain a high-quality bacterial genome skeleton (contig), and then nanopore data was used to connect the high-quality contig into a completed map. For the second-generation sequencing depth statistics, Burrows-Wheeler Aligner [20] (version: 0.7.17) was used to align short illumina sequences to the assembled genome. For the third-generation sequencing depth statistics, Minimap2 [21] (version: 2.11-r797) was used to align the long sequence to the assembled genome. SAMtools [22] (version: 1.9) was used to slide on the genome with a sliding window of 2000 bp. The statistical average sequencing depth reflected the reads coverage of different regions. The average sequencing depths of second-generation and third-generation sequencings were 571.66 X and 365.52 X, respectively.

Prokka [23] (version: 1.1.2) was used to predict the encoded genes of the assembled genome to obtain the gene, CDS, tRNA, rRNA, and tmRNA of the genome. The predicted gene sequences were compared with Kyoto Encyclopedia of Genes and Genomes (KEGG, version: 87.0-r20180701), Clusters of Orthologous Groups of proteins (COG, version: 2014).

### Genome sequence analysis of CQ16Z1 and Y97

Prokka (version: 1.14.6) was used to annotate the whole genomes of CQ16Z1 and Y97. The ANI was calculated by the web server (http://enve-omics.ce.gatech.edu/ani/) [24]. The Prophage Hunter was used to analyze the prophages present in the two strains, provided by the web server (https://pro-hunter.genomics.cn/). Genome-to-Genome Distance Calculator 2.1 (GGDC, https://ggdc.dsmz.de/ggdc.php) was used to estimate the dDDH [25]. Mauve [26] (version: 20150226) was used for the collinearity analysis. BLAST Ring Image Generator [27] (BRIG, version: 0.95) was used to draw the comparison circle map of chromosomes. OrthoFinder [28] (version: 2.5.2) was used to analyze the shared genes and unique genes. Tbtools [29] (version: 1.0971) was used to draw the Venn diagram of the shared genes and unique genes. DIAMOND [30] (version: 2.0.9.147) was used for protein COG database annotation (version: 2020), and BLASTP (version: 2.9.0) was used for protein KEGG database annotation. In addition, eggNOG-Mapper [31] (version: 2.0.1; built-in database version: 5.0.2) was used for further functional annotation of genes. ARDB database was used to predict drug resistance genes [32].

### Resistance of CQ16Z1 and Y97

The unique gene of CQ16Z1 and Y97 showed that there was a large difference in the resistance genes. Therefore, we tested the resistance using 13 antibiotics, including penicillin, ampicillin, daptomycin, vancomycin, linezolid, streptomycin, gentamicin, rifampicin, chloramphenicol, doxycycline, erythromycin, tigecycline and teicoplanin. *Enterococcus faecalis* ATCC29212 was used as the quality control strain. The MA120 bacterial identification and drug sensitivity analysis system and its matching detection board were provided by Zhuhai Meihua Biotechnology Company. The detection method was similar to the previous reports [33], specifically: aseptically select the strains to be identified in the isolated pure culture medium, adjusting the bacterial solution turbidity to 2 Michaelis units in strict accordance with the instructions of the identification instrument, add the bacterial suspension into each well of the reagent strip, 100ul per well, and incubate at 35°C for 24 hours (Individual slow growth can be extended to 36–48 hours). After incubation, the reagent plate was analyzed with MA120 bacterial identification and drug sensitivity analysis system, and the data were collected.

### Open field test (OFT)

The open field test was used to study whether the two strains of bacteria have differences in the exploratory behavior of mice. The experimental mice were divided into three groups: control group, Y97 group and CQ group. Specific experimental methods: preparation of CQ16Z1 and Y97 bacterial solution: referring to the previous study [34], CQ16Z1 and Y97 were resuspended with sterile normal saline at a concentration of 1.0 × 10^9^ CFU/mL. The experimental animals were healthy male C57BL/6 mice, 48 in total, 16 mice in each group, with an initial weight of 20–25 grams, purchased from Chengdu Dossy Experimental Animals Co., Ltd. The method of open field test was similar to the previous report, with a few modifications [35] During the experiment, each mouse was placed in an open field test box (50 cm × 50 cm × 40 cm), the distance and time of mice in the central area within 5 minutes were recorded using video capture software.

The CQ and Y97 groups were intragastrically administered with CQ16Z1 or Y97 bacterial suspension for 28 days at the dose of 100 μL per day. The control group was fed with the same amount of normal saline. Then an open field test was performed on the 29th day, and the data was statistically analyzed. The three groups of mice were kept in separate rooms in the SPF experimental animal room of Chengdu Medical College (temperature: 22–25°C; light: 12:12 h light-dark cycle). During the feeding period, they ate freely (SPF-grade feed, Chengdu Dashuo experiment Animal Co.) and sterile drinking water. All experiments were carried out in accordance with the principles of care and use of laboratory animals approved by the Chengdu Medical College Committee on Welfare Ethics of Laboratory Animals (Approval No: ChengYiDongLun-21-005). Referring to the previous study [36], mice were anesthetized with 4% isophane. After the experiment, the mice were killed by cervical dislocation, and all appropriate measures were employed to minimize suffering.

### Elevated Plus Maze Test (EPM)

The elevated plus maze (EPM) test was performed on the day following the open field test. Experimental methods refer to previous studies [34]. In short, mice were placed at the intersection of the open arm and the closed arm of the elevated plus maze, facing the open arm, and allowed to explore the maze freely for 5 minutes. The number of entries to the open arms and percentage of open arms entry times were utilized as a measure of exploratory behavior. The maze arms were cleaned with 75% ethanol after each use.

### Fecal 16S rRNA Gene V3-V4 region sequencing

The feces of the mice in each group were collected on day 31 and 37, respectively. The collected fresh feces were placed in cryopreservation tubes and sent to Shenzhen Huada Gene Science and Technology Service Co., Ltd. to detect the microbiota through 16S rRNA V3-V4 region amplification.

The sequencing process and data analysis are completed by standard steps. First, the concentration, purity and integrity of the DNA were evaluated by NanoDrop 2000 and agarose gel electrophoresis. Then the library was established by PCR amplification. The fragment range and concentration of the library were detected by Agilent 2100 Bioanalyzer. The qualified library was sequenced according to the size of the inserted fragment. Finally, the hiseq platform was selected for sequencing. After sequencing, the data was filtered to obtain high-quality clean data for later analysis. The reads were spliced into Tags through the overlap relationship between the reads. Usearch [37] (version: 7.0.1090_i86linux32) was used to cluster the spliced Tags with 97% sequence similarity to obtain OTU. The OTU representative sequences were aligned with the Greengene (version: 201305), RDP (version: Release16 20160930), Silva (version: 138), UNITE (version: 8.2 20200220) databases by RDP classifer [38] (version: 1.9.1) for species annotation and classification analysis, etc.

### Metabolomic analysis based on LC-MS/MS

After the feces of mice were taken on the day 31, the mice were anesthetized with 4% isoflurane, and the serum samples of mice in each group were collected through orbital blood collection (6–7 mice in each group). The analysis of metabolites was based on liquid chromatography mass spectroscopy/mass spectroscopy was performed on a vanquish UHPLC system (Thermo Fisher, Germany) coupled with a Q active ™ HF system (Thermo Fisher, Germany). Both positive and negative ion modes were considered.

The screening of differential metabolites mainly refers to FC and *P*-value. VIP refers to the Variable Importance in the Projection of the first principal component of the PLS-DA model, and VIP value refers to the metabolites. Contribution to grouping; FC refers to the fold of difference (FoldChange), which is the ratio of the mean values of all biological repeat quantitative values of each metabolite in the comparison group; *P*-value is calculated by T-test, indicating the significance of the difference level. Thresholds were set at VIP > 1.0, FC > 1.2 or FC < 0.833 and *P* value < 0.05.

### Data processing

SPSS 26.0 was used for data statistical analysis, Graphpad Prism 7.00 was used for plotting data statistical analysis results. Continuous variables were presented as the mean ± standard error of the mean (SEM). Results were analyzed using one-way ANOVA. LSD test and tamhane’s T2 test were used to analyze behavioral data of mice. T-test was chosen for the analyses of metabolomics data. The Kruskal-Wallis test was used to analyze the gut microbiota data. The significance level is α = 0.05.

## Results

### CQ16Z1 genome

**Fig 1:**
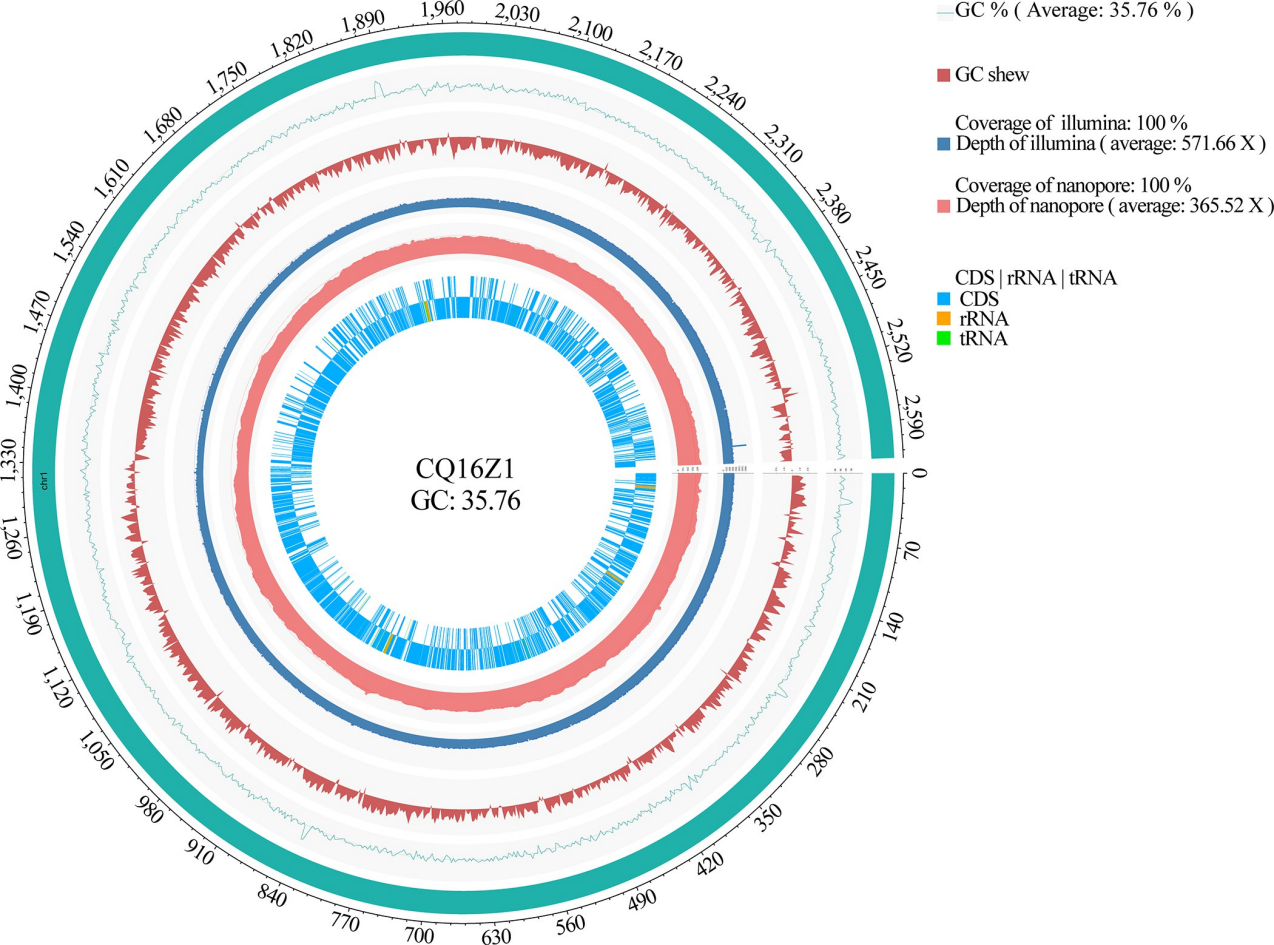
Genome circle of *Companilactobacillus futsaii* subsp. *chongqingii* CQ16Z1.

The whole genome of CQ16Z1 contained 1 chromosome and 5 circular plasmids. The chromosome size was 2,625,188 bp, and the GC content was 35.76% (Fig 1). The sizes of the five circular plasmids were: 13,939 bp, 6,387 bp, 3,699 bp, 3,415 bp and 2,333 bp, respectively, and the corresponding GC content were 37.20%, 35.01%, 37.52%, 37.92% and 35.41%. CQ16Z1 genome has a total of 2,744 genes, including 2,547 coding genes, 57 tRNAs, 12 rRNAs, and 1 tmRNA. The genome had been uploaded to the GenBank database. The chromosome accession number was CP064761, and the accession numbers of plasmids were CP064762, CP064763, CP064764, CP064765 and CP064766.

### Genome function annotation

COG functional classifications: all 2139 genes of CQ16Z1 can be annotated into 23 functional classifications in the COG database. 166 genes were annotated to E (amino acid transport and metabolism, 7.76%), 205 genes were annotated to G (carbon transport and metabolism, 9.58%), 172 genes were annotated to K (transcription, 8.04%), 101 genes were annotated to M (cell wall/membrane/envelope biology, 4.72%), and 192 genes were annotated to X (Mobile: phases, transports, 8.98%), 200 genes were annotated to J (translation, ribosomal structure and biogenesis, 9.35%). 108 genes were annotated to L (replication, recommendation and repair, 5.05%). 101 genes were annotated to P (organic ion transport and metabolism, 4.72%), and the number of other genes classified in COG annotation was less than 100 (S1 Fig).

KEGG functional classifications: there were 2186 genes of CQ16Z1 that can be annotated in the KEGG database. There were 192 genes related to membrane transport, 221 genes related to carbohydrate metabolism, 115 genes related to metabolism of cofactors and vitamins, and 119 genes related to nucleoside metabolism (S2 Fig).

### Genome sequences study of CQ16Z1 and Y97

#### ANI and dDDH of CQ16Z1 and Y97

The average nucleotide identity (ANI) between CQ16Z1 and Y97 genome was 99.20%. The digital DNA-DNA hybridization (dDDH) value was 91.00%.

#### Prophage analysis of CQ16Z1 and Y97

The prophage prediction results showed that Y97 genome contained 9 prophage regions, while CQ16Z1 genome contained 3 prophage regions. No genes related to antibiotic resistance were observed in the prophage region of CQ16Z1, a PBP1A family penicillin-binding protein gene was analyzed in the prephage (*Lactobacillus* phage phiPYB5) contained in Y97.

#### Collinearity analysis results

**Fig 2:**
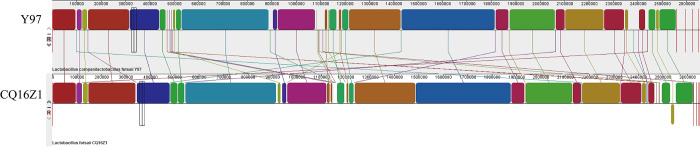
Mauve alignment results of CQ16Z1 and Y97. CQ16Z1 represented *Companilactobacillus futsaii* subsp. *chongqingii* CQ16Z1, and Y97 represented *Companilactobacillus futsaii* subsp. *futsaii* YM0097.

Mauve can be used to effectively identify conserved genomic regions and rearrange regions in multiple genomes. The comparison result showed (Fig 2) that the genomic collinear relationship between CQ16Z1 and Y97 was good. The genome structure of the two strains was basically similar, with a small amount of genetic inversion, for example, between 2.5 Mb and 2.6 Mb.

#### Comparison results of chromosomes circle diagram

**Fig 3:**
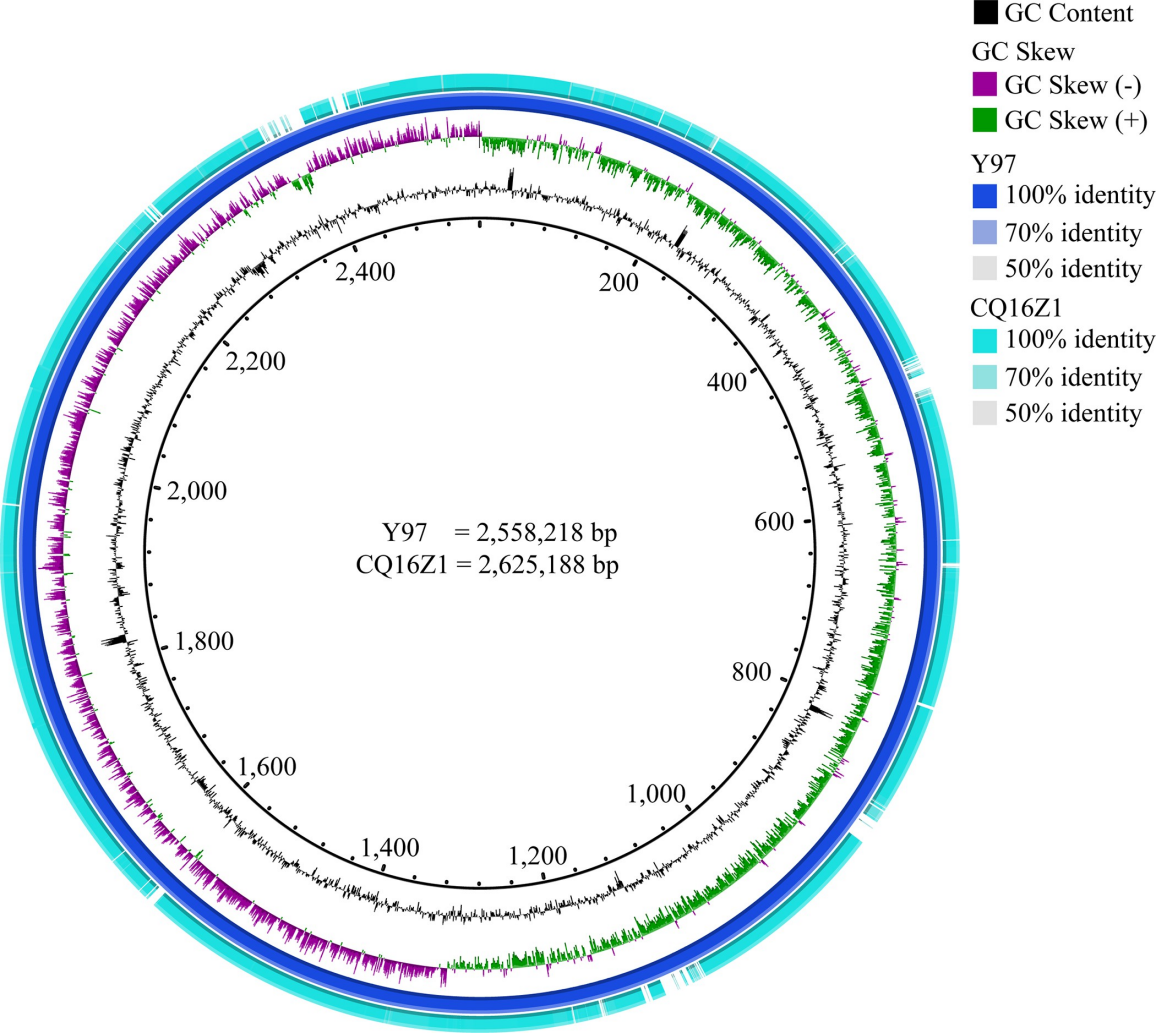
Chromosomes comparison circle of CQ16Z1 and Y97. 1.From the outside to the inside, the first circle represented the chromosome of CQ16Z1, and the second circle represented the chromosome of Y97. 3. The minimum identity was 50%.

The results of BRIG software showed the chromosomes of CQ16Z1 and Y97 were highly similar, with some differences. There were only 7 larger gaps in the circle diagram, where the identity of sequences was less than 50% (Fig 3).

#### Analysis of shared and unique genes

**Fig 4:**
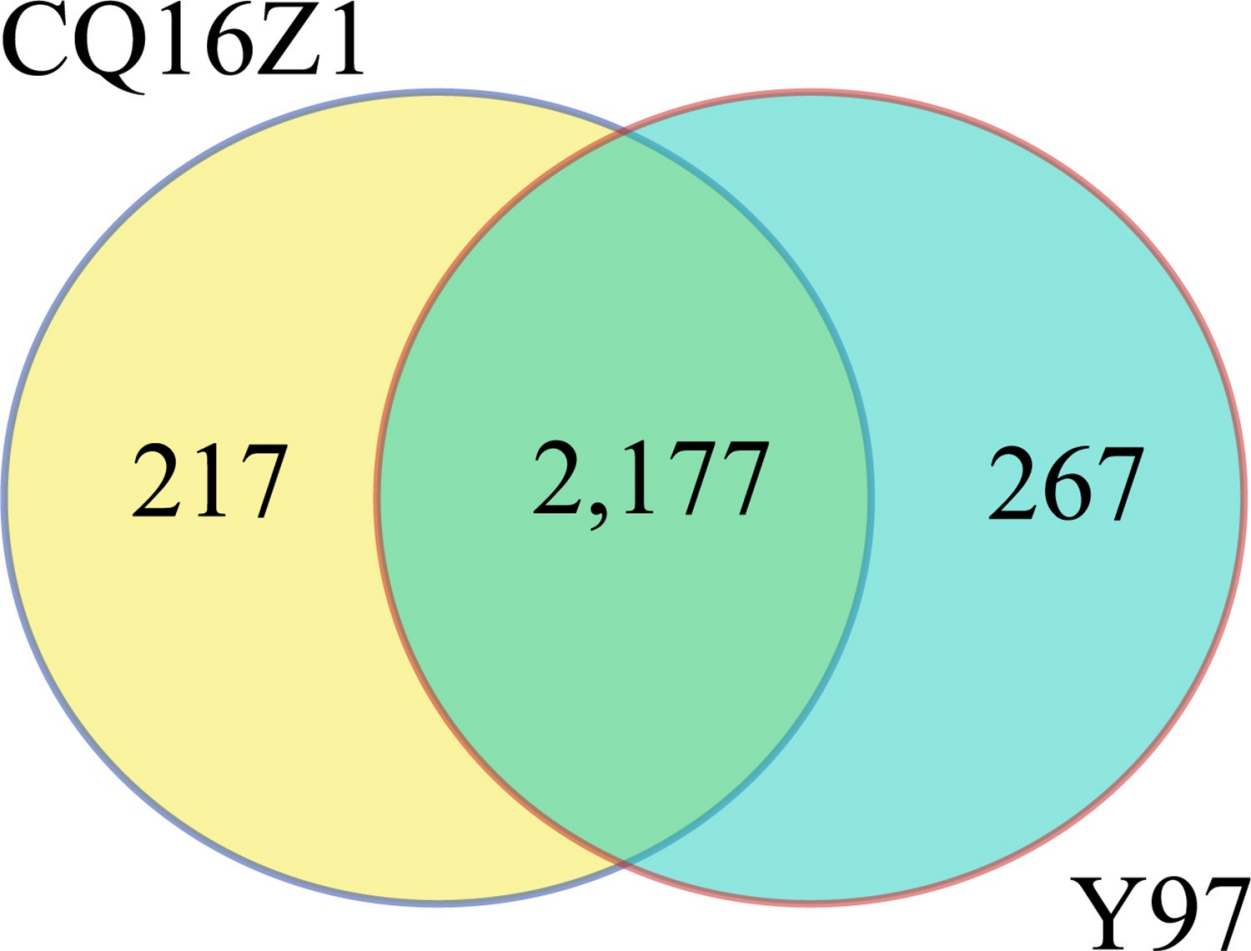
Number of shared genes and unique genes of CQ16Z1 and Y97. 1. = The left part represented unique genes of CQ16Z1, the right part represented unique genes of Y97, and the overlapping part in the middle represents the shared genes of the two strains.

The shared and unique genes of the whole genomes of CQ16Z1 and Y97 were analyzed by OrthoFinder. Shared genes were homologous genes existing in the genomes of both strains, unique genes were genes uniquely owned by one of two genomes. The shared and unique genes can be used as the basis for the study of phenotypic differences. The results showed that there were 2177 shared genes between CQ16Z1 and Y97. CQ16Z1 contained 217 unique genes, 9% of the whole genome, and Y97 contained 267 unique genes, 11% of the whole genome (Fig 4). Most of the unique genes of the two strains were located on the chromosomes.

Unique genes of CQ16Z1 and Y97 were annotated into KEGG and COG databases by BLASTP and DIAMOND, respectively. Among the unique genes of CQ16Z1 and Y97, 95 and 106 genes were respectively provided with functional annotations in the COG database, which mainly annotated to G (carbohydrate transport and metabolism), K (transcription) and L (replication, recombination and repair), V (defense mechanisms) and other classifications (S3 and S4 Figs).

37 and 33 unique genes of CQ16Z1 and Y97, respectively, were annotated into KEGG databases, which were mainly related to classifications such as membrane transport, carbohydrate metabolism, energy metabolism, biosynthesis of other secondary metabolites (S5 and S6 Figs).

To further obtain the functional information of the unique genes, the eggNOG-Mapper (parameter default) was used. The results showed that 174 and 205 unique genes were annotated for CQ16Z1 and Y97, respectively. First, Y97 was obviously more phage-related genes than CQ16Z1. Second, Y97 contained some genes that may be related to drug resistance, which were not annotated in CQ16Z1. Third, Y97 had 5 phosphotransferase system (PTS) related genes, while CQ16Z1 contained only 1. Finally, there were some genes related to rhamnose metabolism in CQ16Z1, such as L-rhamnose isomerase, Y97 had not (S1 and S2 Files).

Classifications of unique genes with obvious differences between two strains (Table 1). The number of genes related to environmental information processing, cofactor and vitamin metabolism, cell wall/membrane/envelope biogenesis were different, which may affect the probiotics of the strain. Y97 contained more resistance genes than CQ16Z1, suggesting that Y97 had more extensive drug resistance. There were 29 phage-related genes in Y97, and only 8 in CQ16Z1, and the types were different. For example, phage minor structural protein GP20 and the tail tape measure protein TP901 only existed in Y97. Although both strains contained phage terminase gene, the identity was only 26.32%. In addition, CQ16Z1 contained more genes related to carbohydrate metabolism.

**Table 1:** List of differences in the number of CQ16Z1 and Y97 unique genes based on fuanctional classification.

Classifications	G		E	V	M	P	R
Databases	COG	KEGG	KEGG	KEGG	COG	eggNOG	eggNOG
CQ16Z1	17	11	4	0	1	8	0
Y97	9	7	5	6	2	29	7

G: Carbohydrate metabolism, E: Environmental information processing, V: Metabolism of cofactors and vitamins, M: Cell wall/membrane/envelope biogenesis, P: Phage related genes, R: Drug resistance related genes.

### The resistance of CQ16Z1 and Y97

The resistance results showed that there were significant differences in the MIC values of 6 of 13 antibiotics between Y97 and CQ16Z1. Among them, the difference of MIC value of penicillin was the largest, 64 times. In general, Y97 showed different degrees of resistance to 13 antibiotics, while CQ16Z1 was sensitive to more than half of them (Table 2).

**Table 2:** The MIC values of CQ16Z1 and Y97.

Antibiotic	CQ16Z1 (μg/mL)	Y97 (μg/mL)
Penicillin	0.25	≥16
Ampicillin	1	≥16
Daptomycin	4	≥8
Vancomycin	≥32	≥32
Linezolid	≤1	≥8
Streptomycin	≤1000	>1000
Gentamicin	≤500	>500
Rifampin	>4	>4
Chloramphenicol	≥32	≥32
Doxycycline	0.5	≥16
Erythromycin	≥8	≥8
Tigecycline	≤0.25	≥1
Teicoplanin	≥32	≥32

MIC was a range value of antibiotic concentration.

We analyzed the possible drug resistance genes in the whole genomes of the two strains. Both strains contained some genes involved in drug resistance, such as vancomycin resistance related genes *vanA* and *vanB*, ribosomal protection protein gene (*tetM*), penicillin binding protein genes (PBPs), chloramphenicol acetyltransferase gene (*cat*) and ABC transporters. In addition, Y97 contained glycopeptide antibiotic resistance related genes *vanre*, macrocyclic propylester antibiotic resistance gene *msrA*, and multidrug resistance efflux protein resistance genes *mdtg* and *emea*.

### The effect of CQ16Z1 and Y97 on the exploratory behavior of mice

**Fig 5:**
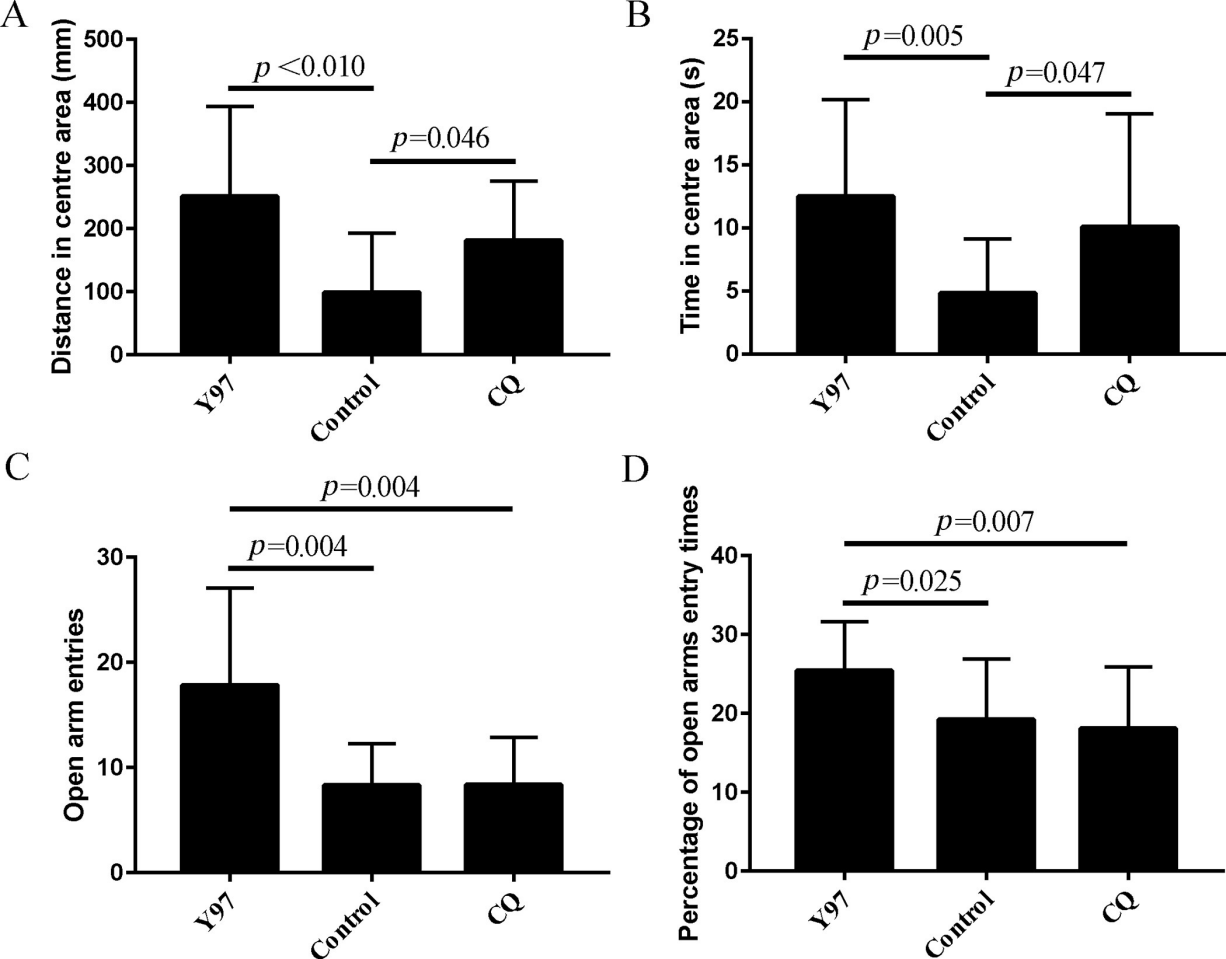
Behavioral experimental results of three groups of mice. 1. CQ represented mice fed with *Companilactobacillus futsaii* subsp. *chonqingii* CQ16Z1, Y97 represented mice fed with *Companilactobacillus futsaii* subsp. *futsaii* YM0097. 2. A showed the distance in centre area, and B showed the time in centre area, C showed the number of entries to the open arms and D showed the percentage of open arms entry times. 3. Data were presented as mean ± SEM. One-way ANOVA followed by LSD test and Tamhane’s T2 test. The significance level was α = 0.05. (n = 14–16 mice per group).

In the OFT, significant differences were observed between groups for the distance in centre area (mean±SEM =: Y97: 250.62±35.680; CQ: 180.56±23.800; control: 98.53±23.653). One-way ANOVA revealed a significant difference in distance [one-way ANOVA, F (2, 45) = 7.246, *P* = 0.002]. Multiple comparisons between groups showed that there were differences in Y97 and CQ compared with the control group (LSD test, *P* < 0.010; *P* = 0.046) (Fig 5A). Compared with the control group, the time in centre area was also different (mean±SEM =: Y97: 12.47±1.930; CQ: 10.05±2.244; control: 4.80±1.083). One way ANOVA showed significant differences in time [F (2, 45) = 4.645, *P* = 0.015]. Y97 group and CQ group were significantly different from the control group (LSD test, *P* = 0.005; *P* = 0.047) (Fig 5B). There was no significant difference between Y97 and CQ group, whether it was the time in the centre area or the distance in the centre area.

In the EPM, significant differences were observed between groups for the number of entries to the open arms (mean±SEM =: Y97: 18.29±2.575; CQ: 8.79±1.163; control: 8.29±1.061). [one-way ANOVA, F (2, 42) = 10.943, *P* < 0.010]. Multiple comparisons between groups showed that there was significant difference between Y97 group and control group, and it was also significantly different from CQ group, while no significant difference was found between CQ group and control group (Tamhane’s T2 test, *P* = 0.004; *P* = 0.004) (Fig 5C). Similar results were shown in the percentage of open arms entry times for the three groups (mean±SEM =: Y97: 25.68±1.761; CQ: 18.22±2.179; control: 19.21±2.057). [one-way ANOVA, F (2, 42) = 4.588, *P* = 0.016]. (LSD test, *P* = 0.025; *p* = 0.007) (Fig 5D).

The above results showed that Y97 was beneficial to the exploratory behavior of mice, and suggesting that Y97 was more likely to be a psychobiotics.

#### Analysis of fecal samples

**Fig 6:**
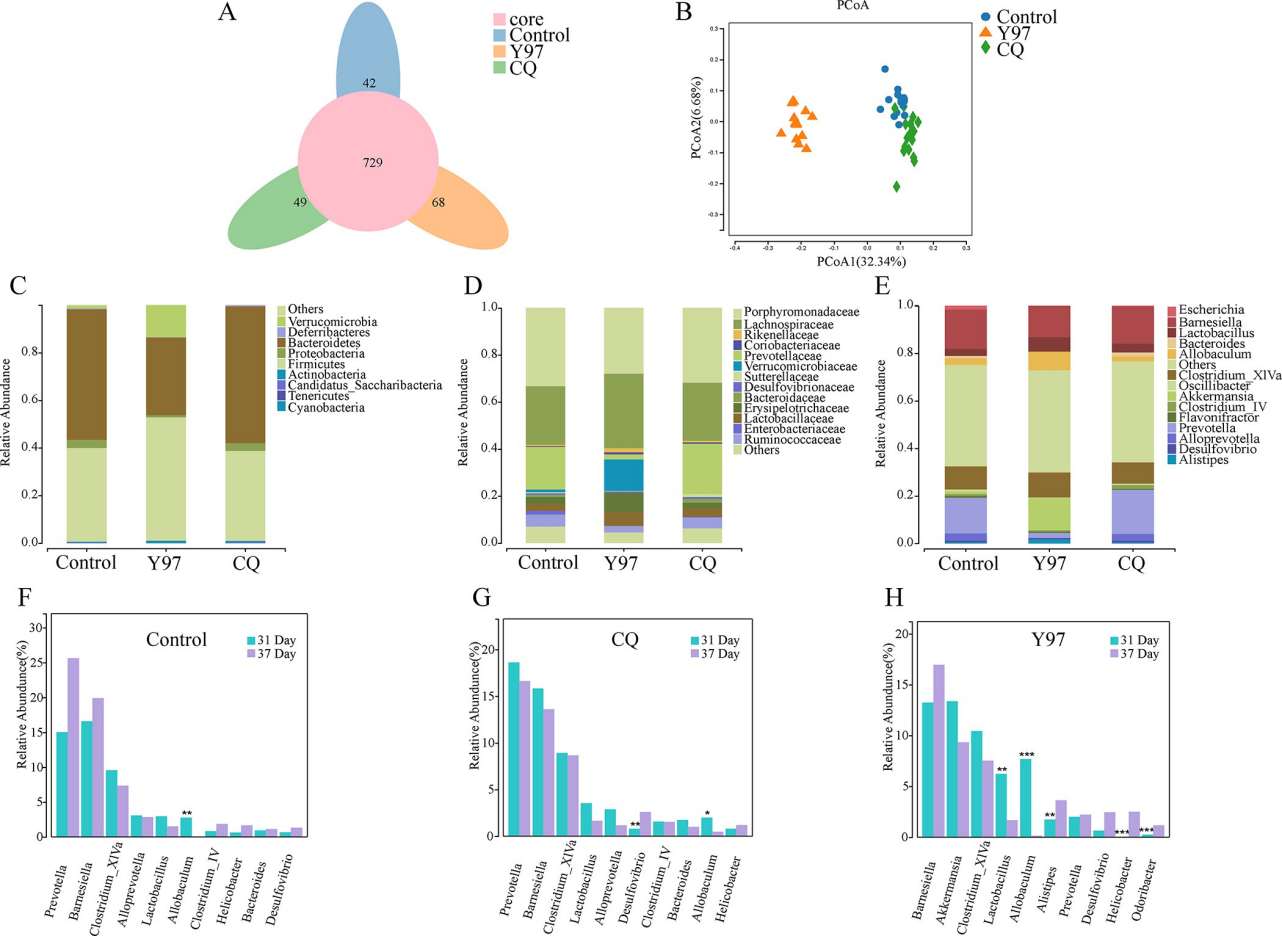
Results of the gut microbiota of three groups of mice. 1. CQ represented mice fed with *Companilactobacillus futsaii* subsp. *chonqingii* CQ16Z1, Y97 represented mice fed with *Companilactobacillus futsaii* subsp. *futsaii* YM0097. 2. A. Petal diagram of OTUs. Each circle of different colors represented different groups. The number of overlapped parts within the circle indicates common OTUs among the groups, and the number outside the overlapping parts indicates the unique OTUs in each sample group. B. PCoA analysis. Different colors in the graph represented different groups of samples. A point was a sample. The closer the points were, the more similar the samples were, otherwise, the greater the difference was. Fig 6F-6H compared the changes of gut microbiota at the genus level in the three groups of mice on the day 31 and 37, respectively. The abscissa was the sample name, and the ordinate was the relative abundance of the annotated species. Species that were not annotated at this taxonomic level and whose abundance was less than 0.5% in the sample were merged into Others.

The OTU clustering Petal diagram could analyze the common and unique OTUs between different groups. The Y97 group, CQ group, and control group had 729 OTUs in common, and their unique OTUs were 68, 49, and 42, respectively (Fig 6A). Beta diversity analysis was mainly used to compare differences in species diversity between samples. Beta diversity analysis was generally represented using a PCoA plot. PcoA analysis was performed based on the UnWeighted_UniFrac distance. The samples in Y97 group were located in different regions from those in CQ group and control group. The microbiota of Y97 group was farther away from the two groups, which was different (Fig 6B).

To further understand the link between the gut microbiota and the strains studied, we performed species-relative abundance assessments at the phylum, family, and genus levels of the gut microbiota in different groups of mice. Fig 6C showed the relative abundance map of the three groups of mice at the phylum level on the day 31, Fig 6D was the family level, and Fig 6E was the genus level. At the phylum level, *Firmicutes and Bacteroidetes* were the main dominant bacterial phylum in three groups, and except *Firmicutes*, Y97 group had significant differences compared with control group (*P*<0.05). In these categories, there was no significant difference between CQ group and control group (*P*>0.05) (S3 File). At the family level, compared with the control group, feeding Y97 increased the relative abundance of *Rikenellaceae*, *Lachnospiraceae*, *Lactobacillaceae*, *Erysipelotrichaceae* and *Verrucomicrobiaceae* in the intestinal flora of mice, and decreased the relative abundance of *Ruminococcaceae*, *Sutterellaceae* and *Prevotellaceae* in the intestinal flora. With the exception of *Lachnospiraceae*, there were significant differences among these families (*P* values were less than 0.05). However, the differences in these categories were not statistically significant between CQ group and control group (S4 File). At the genus level, the relative abundances of *Alistipes*, *Akkermansia*, *Allobaculum* and *Lactobacillus* in Y97 group were higher than control group, while the relative abundances of *Prevotella* and *Oscillibacter* were lower than control group (*P* values were all less than 0.05). In terms of these genus categories, there was no significant difference between CQ group and control group (S5 File). Feeding Y97 significantly altered the gut microbiota relative to the control and CQ groups.

Fig 6F showed the histogram of key species at the genus level compared between the controls of day 31 and 37, Fig 6G was comparison between CQ groups, and Fig 6H was the comparison between Y97 groups. One genus (*Allobaculum*, *P*<0.05) was significantly different in control group (S6 File). In CQ group, there were 2 genera with a significant difference (*Desulfovibrio* and *Allobaculum*; *P*<0.05) (S7 File). In Y97 group, 5 significant differences were found (The relative abundance of *Lactobacillus* and *Allobaculum* decreased, *Alistipes*, *Helicobacter* and *Odoribacter* increased; *P*<0.05) (S8 File). At the family level. in Y97 group (comparison of day 31 and 37), *Erysipelotrichaceae*, *Lactobacillaceae*, *Rikenellaceae*, *Helicobacteraceae and Porphyromonadaceae* were significantly different (*P*<0.05). When the bacteria breeding had been stopped for one week, the intestinal flora of Y97 group showed the most obvious change, which indicated that Y97 had a stronger effect on the intestinal flora than CQ16Z1.

### Metabolomic signatures of mice

In the positive ion mode, compared with the control group, a total of 52 and 107 metabolites with significant differences were screened out in Y97 group and CQ group (S9 and S10 Files, S7 Fig), and 67 and 57 metabolites with significant differences were screened out in the negative ion mode (S11 and S12 Files, S8 Fig). Referring to previous studies, we analyzed some indicators of positive correlation (cortisol and 4-hydroxybenzoic acid) and negative correlation (5-hydroxyindole-3-acetic acid, kynurenic acid, 3-indoleacrylic acid and hippuric acid) with depression or anxiety [8–11]. Between CQ group and control group, there were 5 significant differences (*P*<0.05), but the cut in cortisol was not conducive to the relief of depressive symptoms. Between Y97 group and control group, 3 indicators had statistical differences(*P*<0.05), and were all conducive to the relief of depression. In addition, of the 6 indicators surveyed, only one showed equal significance in both groups (Table 3).

**Table 3:** Some differential metabolites in serum of three groups.

Metabolites	CQ-vs-Control			Y97-vs-Control		
	FC	*P*-value	Down-or-Up	FC	*P*-value	Down-or-Up
cortisol	1.952	0.019	up	1.573	0.042	down
4-Hydroxybenzoic acid	0.633	0.003	down	1.488	0.006	down
5-Hydroxyindole-3-acetic acid	1.318	0.328	-	0.453	0.013	up
kynurenic acid	2.295	0.002	up	0.552	0.284	-
3-indoleacrylic acid	1.561	0.002	up	0.738	0.052	-
hippuric acid	1.934	0.037	up	0.825	0.350	-

## Discussion

In this study, we obtained the whole genome sequence of CQ16Z1, which can be used for genome sequence analysis with its related subspecies Y97. Collinearity analysis, circle diagram comparison, the ANI value and dDDH value supports that they are closely related and belong to *Companilactobacillus futsaii*.

In the analysis of unique genes and shared genes, the ratio of unique genes is greater than 10%. Unique genes lead to phenotypic differences between the two bacteria, and also reveal that the two bacteria have experienced different evolutionary events. Our previous research showed that CQ16Z1 can utilize more sugars such as gluconate, galactose and ribose and its cell well contained more sugar components than Y97 [7]. The differences may be due to the fact that CQ16Z1 has more G (carbohydrate transport and metabolism) genes than Y97, such as sugar (pentulose or hexulose) kinase and Glycogen debranching enzyme (alpha-1,6-glucosidase), and these genes are all located on the chromosomes. In addition, neither strain can metabolize L-rhamnose [7], which may be due to gene silencing of L-rhamnose genes of CQ16Z1.

Y97 has more phage related genes and prophages, and the type of phage genes are different between the two strains. Phage-mediated horizontal gene transfer may play an important role for non-pathogenic bacteria in adapting to specific environments [39], and can promote the evolution of microorganisms and increase the diversity of host communities [40,41]. Studies have reported that the number of prophages in different subspecies is also significantly different. For example, in *Streptococcus phocaeas* subsp. *phocae* and *Streptococcus phocaeas* subsp. *Salmonis*, the number of prophages contained in them are 8 and 2, respectively. The differences in these phages may constitute the genetic basis for their pathogenicity [42].

The research on drug resistance of probiotics has always attracted much attention [18]. In most organisms, ABC transporters constitute one of the largest membrane protein families, and ABC transporters may have important significance in drug resistance [43]. The presence of transporters makes certain bacteria inherently resistant to antibiotics, which allows them to eliminate antibiotics before they cause cell damage. These transporters may only target one molecule or a few structurally unrelated molecules, such as the ABC-type multidrug efflux system of *Bifidobacterium longum* [44,45]. And the ABC type multidrug transport system, ATPase component increased the minimum inhibitory concentration of *Enterococcus faecium* on the polyther ionophore antibiotic Narasin [46]. Consistent with previous reports, the ribosomal protection protein gene (*tetM*) and the chloramphenicol acetyltransferase gene (*cat*) present in *Lactobacillus* mediate bacterial resistance to tetracycline and chloramphenicol, and The MICs of *Lactobacillus* against penicillin, ampicillin, and linezolid were also consistent with our study, but in this study the MIC of *Lactobacillus* against rifampicin and vancomycin was higher than our results [47]. *Mdtg* and *emea* are responsible for multidrug resistance in *Salmonella enterica* subsp. *enterica serovar Typhi* and *Enterococcus*, which are considered as multidrug resistance efflux proteins [48,49]. Y97 showed resistance to a range of antibiotics, which may be limiting for Y97 to be studied as a probiotic.

*Lactobacillus*, as a common probiotic, has been reported to have multiple benefits [50]. In our study, Y97 and CQ16Z1 had significant differences in the number of genes of environmental information processing, membrane transport, metabolism of cofactors and vitamins and cell wall/membrane/envelope biogenesis. These differences may make Y97 more adaptable to intestinal environment and better able to synthesize products that are beneficial to the host. Studies have shown that *L*. *crispatus* from different sources has different adaptability to the environment, such as fecal-derived strains and vagina-derived strains, and across the two niches, genes associated with membrane transport (~11%) occupied a large proportion [51]. In addition, the unique genes of Y97 contain more phosphotransferase system (PTS) related genes. It has been reported that the PTS gene can make *Lactobacillus johnson*ii NCC533 possess a longer gut persistence [52]. Vitamins also play an important role in the probiotic effects of probiotics, such as vitamin B_12_ [53]. Although the whole genomes of CQ16Z1 and Y97 contain vitamin B_12_-related genes, in the unique gene classification, only Y97 has. At present, there are many studies on the application of *Lactobacillus* as a probiotic in psychiatric diseases, mainly focusing on the transmission pathway from the gut to the brain and the mechanism of its impact on the gut flora. For example, a probiotic-supplemented diet containing *Bifidobacterium* and *Lactobacillus* acidophilus alleviates cognitive behavior in a mouse model of Alzheimer’s disease [54]. Probiotic preparations containing *Lactobacillus plantarum* Lp3, *Lactobacillus rhamnosus* LR5, *Bifidobacterium lactis* BL3, *Bifidobacterium breve* BR3, and *Pediococcus pentosaceus* PP1 may improve depression by altering the composition of the gut microbiota [55]. Adding probiotics to fermented milk for two weeks improved the exploratory activity and anxiety behavior of elderly mice [56]. In addition, the open field test and the elevated plus maze test play an important role in assessing mouse behavior. For example, the elevated plus maze can be used to detect anxiety-like behaviors in mice [57]. Feeding *Lactobacillus plantarum* PS128 significantly increases the total distance traveled in the open field test and reduces the amount of time spent in closed arms in the elevated plus maze test [58].

From the analysis of OTU clustering and beta diversity, Y97 has a certain effect on intestinal flora. *Bacteroidetes* and *Firmicutes* were the main Phylum of the three groups of mouse intestinal flora, but the relative abundance of *Bacteroidetes* in the Y97 group was the lowest and *Firmicutes* with the highest relative abundance. Studies have shown that the *Firmicutes*/*Bacteroidetes* phylum ratio is positively correlated with gut health [59]. *Akkermansia*, *Allobaculum Alistipes* and *Rikenellaceae*, *Akkermansia*, *Allobaculum* and *Alistipes* may have beneficial effects on the health of the host [60–66]. Our findings demonstrate that Y97 has a significant effect on the intestinal flora of mice, can have a positive effect on the beneficial flora.

Previous studies have shown that 5-HIAA can be decomposed by serotonin intracellular monoamine oxidase (MAO), and both metabolites are closely related to depression and are expressed at low levels in patients with depression [67,68]. In addition, cortisol plays several key roles in promoting homeostasis, including regulating and suppressing healthy stress responses [69]. Jia et al. detected cortisol in the serum of 89 men, and found that cortisol is also expressed at a high level in patients with depression, and believed that cortisol in serum can be used to evaluate patients with depression and non-depression patients [70]. Although several metabolites related to depression and anxiety have been reported, it seems that cortisol has also attracted much attention. A meta analysis of 354 studies found that cortisol has a greater impact on melancholic depressed subjects [71]. In our results, Y97 and CQ16Z1 have opposite effects on cortisol in serum. The Y97 group with significantly lower cortisol showed an improvement in exploratory behavior in behavioral experiments.

In conclusion, the study conducted a correspondence analysis from the bacterial genome to the phenotype. From the perspective of unique genes, the two bacteria have obvious differences in carbohydrate metabolism, phage infection events, drug resistance, the impact on mice’s exploratory behavior, intestinal flora, and the relative content of metabolites in serum. We fail to clearly point out the genes that the Y97 is beneficial to explore behavior. In the future, we will find the key genes that the Y97 plays a psychobiotic role through gene knockout technology.

## Supporting information

S1 Fig COG functional classifications of CQ16Z1. (DOCX)

S2 Fig KEGG functional classifications of CQ16Z1. (DOCX)

S3 Fig COG functional classifications of unique genes of CQ16Z1. (DOCX)

S4 Fig COG functional classifications of unique genes of Y97. (DOCX)

S5 Fig KEGG functional classifications of unique genes of CQ16Z1. (DOCX)

S6 Fig KEGG functional classifications of unique genes of Y97. (DOCX)

S7 Fig Cluster heat map of total differential metabolites in positive ion mode. (DOCX)

S8 Fig Cluster heat map of total differential metabolites in negative ion mode. (DOCX)

S1 File Supplementary Excel 1_CQ16Z1_annotations. (ANNOTATIONS)

S2 File Supplementary Excel 2_Y97_annotations. (ANNOTATIONS)

S3 File Supplementary Excel 3_ phylum level on day 31. (XLSX)

S4 File Supplementary Excel 4_ family level on day 31. (XLSX)

S5 File Supplementary Excel 5_ genus level on day 31. (XLSX)

S6 File Supplementary Excel 6_Compared Control of day 31 and 37. (XLSX)

S7 File Supplementary Excel 7_Compared CQ of day 31 and 37. (XLSX)

S8 File Supplementary Excel 8_Compared Y97 of day 31 and 37. (XLSX)

S9 File Supplementary Excel 9_Y97.vs.Control_positive_annotation. (XLS)

S10 File Supplementary Excel 10_CQ.vs.Control_positive_annotation. (XLS)

S11 File Supplementary Excel 11_CQ.vs.Control_negative_annotation. (XLS)

S12 File Supplementary Excel 12_Y97.vs.Control_negative_annotation. (XLS)
