# Supplementary figures and images for: Whole genome sequence analysis of two subspecies of Companilactobacillus Futsaii and experimental verification of drug resistance and effect on the exploratory behavior of mice based on unique gene
Source: PLoS One. 2022 Sep 9;17(9):e0274244. doi: 10.1371/journal.pone.0274244 (PMC9462788; doi:10.1371/journal.pone.0274244)

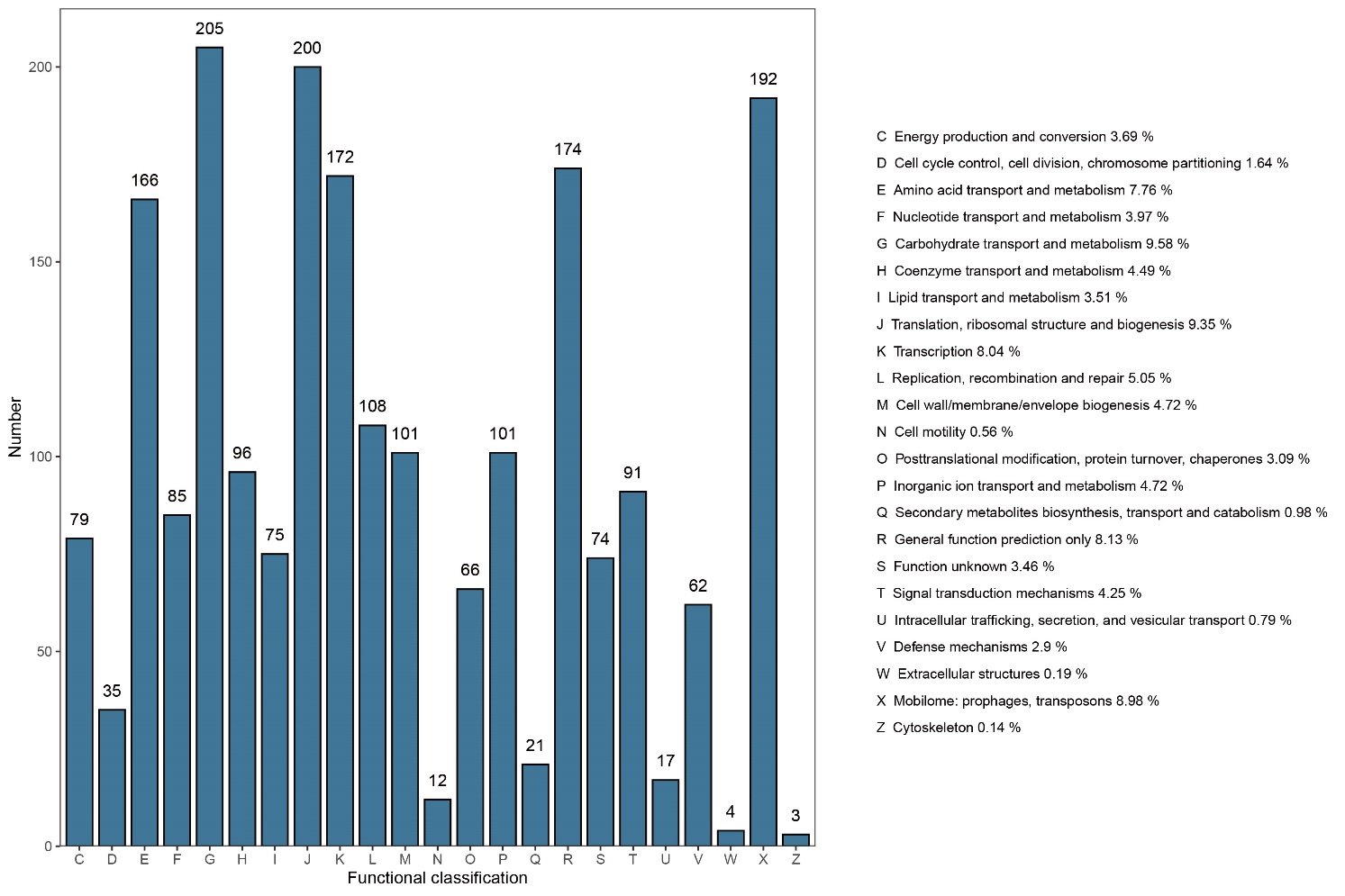


Supplementary Fig S1. COG functional classifications of CQ16Z1.

Supplement: S1 Fig — (DOCX) [file pone.0274244.s001.docx]

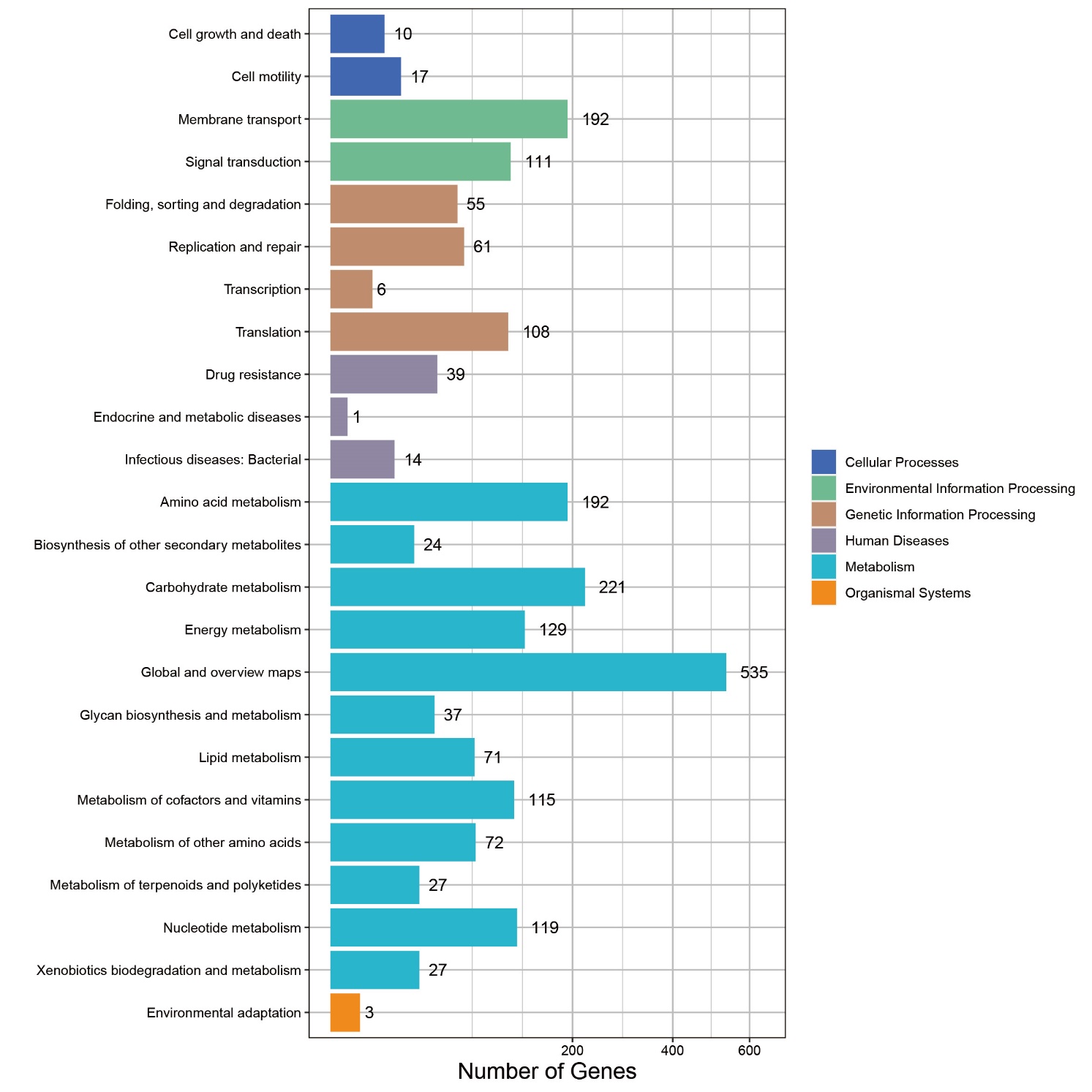


Supplementary Fig S2. KEGG functional classifications of CQ16Z1.

Supplement: S2 Fig — (DOCX) [file pone.0274244.s002.docx]

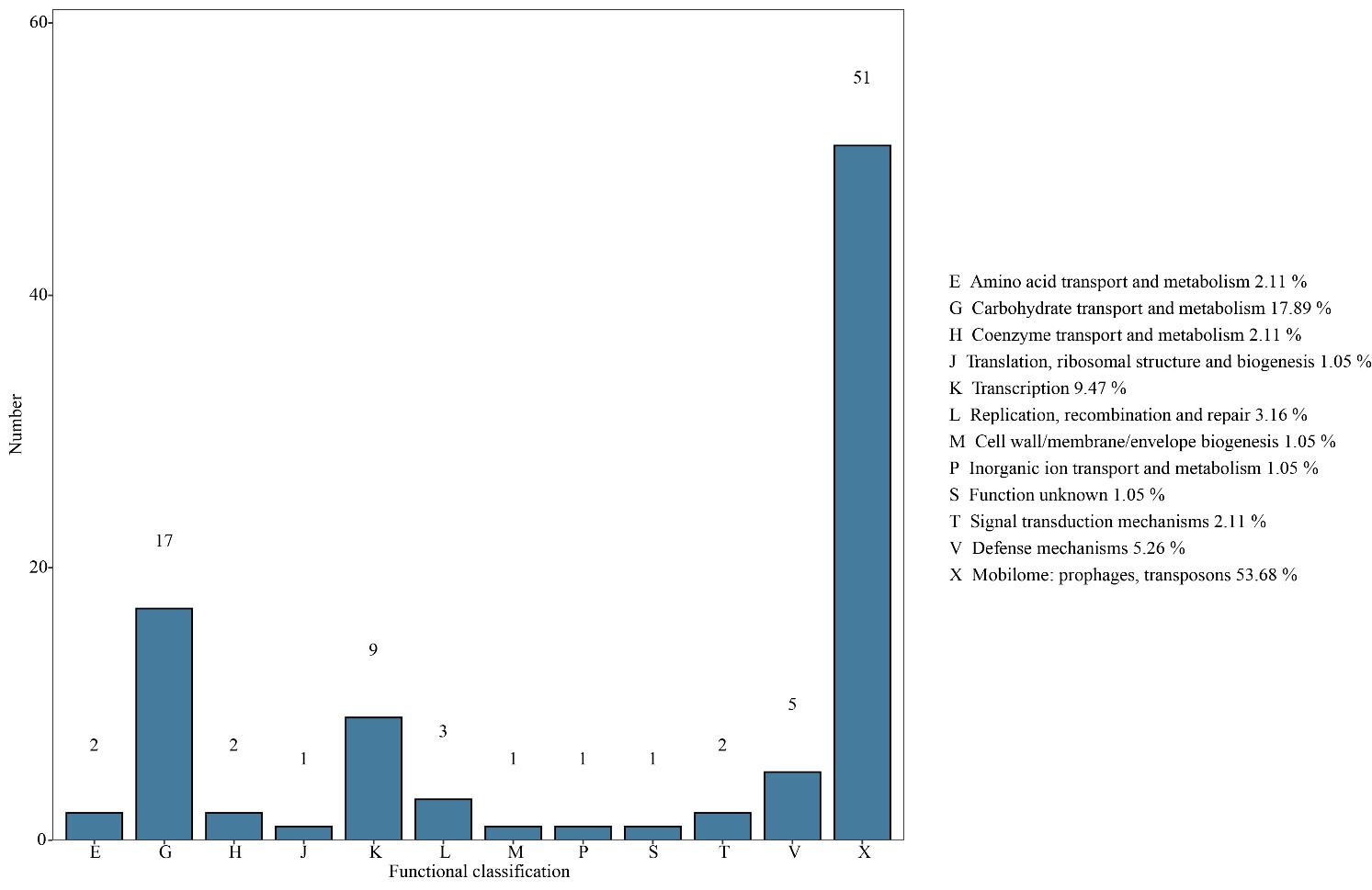


**Supplementary Fig S3.** COG functional classifications of u**nique genes of CQ16Z1.**

Supplement: S3 Fig — (DOCX) [file pone.0274244.s003.docx]

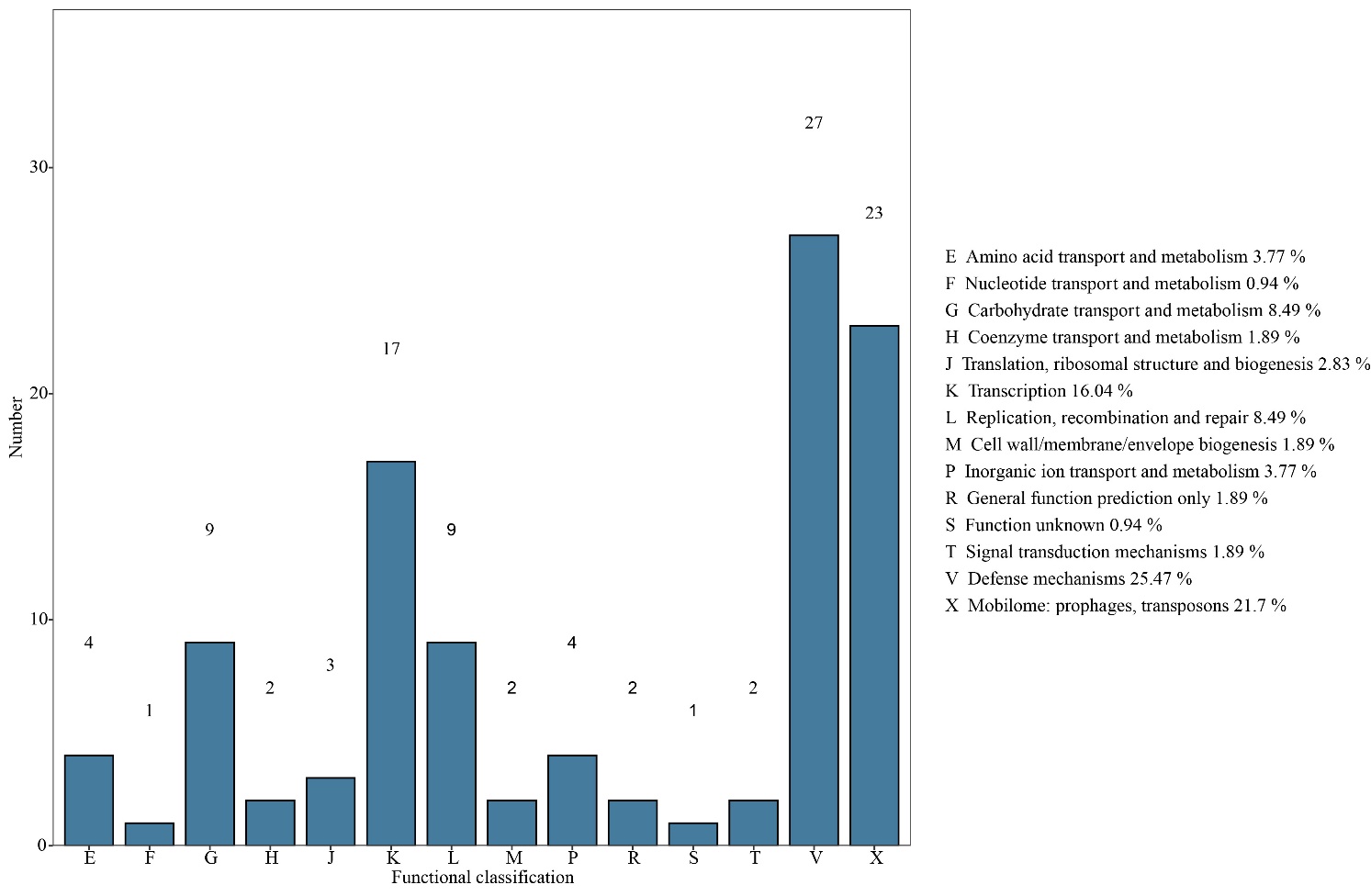


**Supplementary Fig S4. COG functional classifications of unique genes of Y97.**

Supplement: S4 Fig — (DOCX) [file pone.0274244.s004.docx]

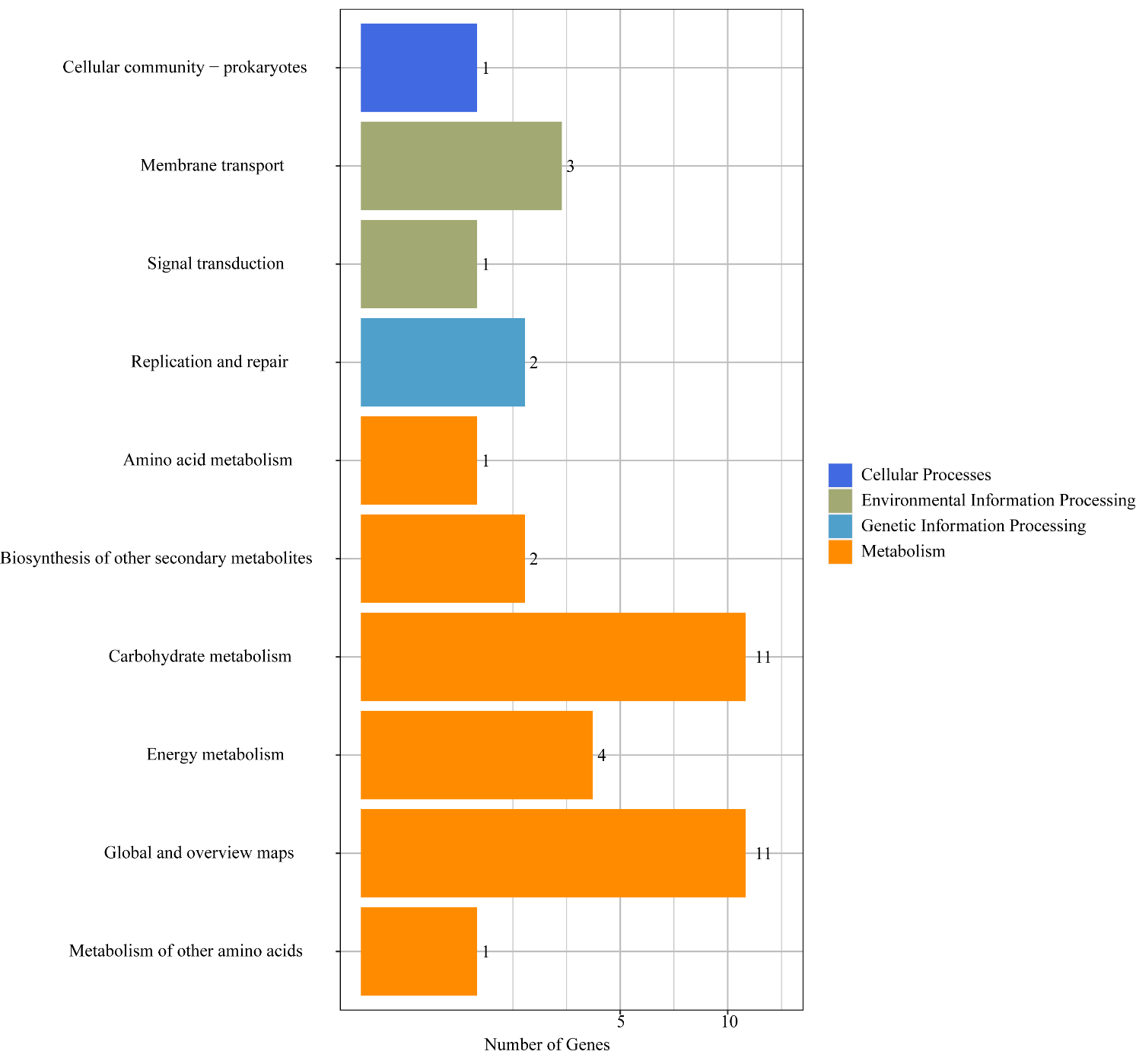


**Supplementary Fig S5.** KEGG functional classifications of **unique genes of** CQ16Z1.

Supplement: S5 Fig — (DOCX) [file pone.0274244.s005.docx]

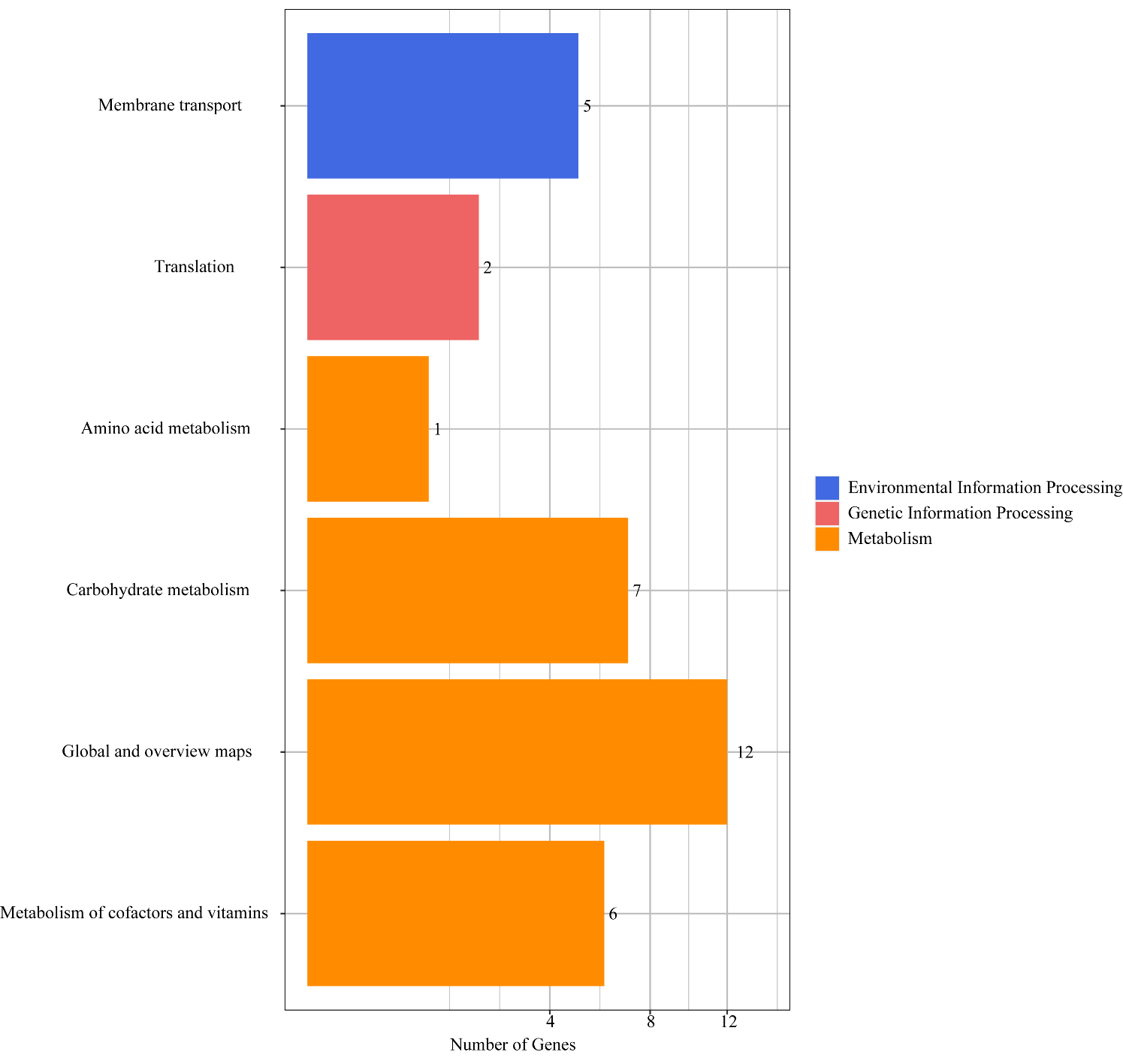


**Supplementary Fig S6.** KEGG functional classifications of **unique genes** of Y97.

Supplement: S6 Fig — (DOCX) [file pone.0274244.s006.docx]
