# Supplementary material for: Whole genome sequence analysis of two subspecies of Companilactobacillus Futsaii and experimental verification of drug resistance and effect on the exploratory behavior of mice based on unique gene
Source: PLoS One. 2022 Sep 9;17(9):e0274244. doi: 10.1371/journal.pone.0274244 (PMC9462788; doi:10.1371/journal.pone.0274244)
Supplement: S7 Fig — (DOCX) [file pone.0274244.s007.docx]

**Supplementary Fig S7.** Cluster heat map of total differential metabolites in positive ion mode
